# Reporter Assay for Endo/Lysosomal Escape of Toxin-Based Therapeutics

**DOI:** 10.3390/toxins6051644

**Published:** 2014-05-22

**Authors:** Roger Gilabert-Oriol, Mayank Thakur, Benedicta von Mallinckrodt, Cheenu Bhargava, Burkhard Wiesner, Jenny Eichhorst, Matthias F. Melzig, Hendrik Fuchs, Alexander Weng

**Affiliations:** 1 Institute of Laboratory Medicine, Clinical Chemistry and Pathobiochemistry, Charité–Universitätsmedizin Berlin, Campus Virchow-Klinikum, Augustenburger Platz 1, Berlin D-13353, Germany; E-Mails: roger.gilabert-oriol@charite.de (R.G.-O.); benedicta.von-mallinckrodt@charite.de (B.M.); cheenu.bhargava@charite.de (C.B.); hendrik.fuchs@charite.de (H.F.); 2 Leibnizinstitut für Molekulare Pharmakologie (FMP), Berlin D-13125, Germany; E-Mails: wiesner@fmp-berlin.de (B.W.); eichhorst@fmp-berlin.de (J.E.); 3 Institute of Pharmacy, Freie Universität Berlin, Königin-Luise-Straße 2 + 4, Berlin D-14195, Germany; E-Mail: melzig@zedat.fu-berlin.de; 4 Wolfson Centre for Gene Therapy of Childhood Disease, University College London–Institute of Child Health, London, 30 Guilford Street, London WC 1N 1EH, UK

**Keywords:** endosomal escape, reporter assay, protein therapeutics, ribosome inactivating proteins, horseradish peroxidase, Alexa Fluor 488, ricin A-chain, saporin, triterpenoidal saponins

## Abstract

Protein-based therapeutics with cytosolic targets are capable of exhibiting their therapeutic effect once they have escaped from the endosomes or lysosomes. In this study, the reporters—horseradish peroxidase (HRP), Alexa Fluor 488 (^Alexa^) and ricin A-chain (RTA)—were investigated for their capacity to monitor the endo/lysosomal escape of the ribosome-inactivating protein, saporin. The conjugates—saporin-HRP, ^Alexa^saporin and saporin-KQ-RTA—were constructed, and the endo/lysosomal escape of these conjugates alone (lack of endo/lysosomal release) or in combination with certain structurally-specific triterpenoidal saponins (efficient endo/lysosomal escape) was characterized. HRP failed in reporting the endo/lysosomal escape of saporin. Contrastingly, Alexa Fluor 488 successfully allowed the report of the process at a toxin concentration of 1000 nM. In addition, single endo/lysosome analysis facilitated the determination of the amount of ^Alexa^saporin released from each vesicle. RTA was also successful in reporting the endo/lysosomal escape of the enzymatically inactive mutant, saporin-KQ, but in this case, the sensitivity of the method reached a toxin concentration of 10 nM. In conclusion, the simultaneous usage of Alexa Fluor 488 and RTA as reporters may provide the possibility of monitoring the endo/lysosomal escape of protein-based therapeutics in the concentration range of 10–1000 nM.

## 1. Introduction

Endo/lysosomal escape is a key step for protein-based therapeutics that exhibit their effect in the cytosol [1]. In general, these kinds of therapeutics are internalized in the cell by a process of endocytosis, which consists of the formation of invaginations in the cell membrane and the subsequent creation of vesicles, called endosomes, that entrap the protein [2]. Endosomes thereafter mature as late endosomes and, finally, to lysosomes. The internal conditions of these vesicles are regulated at each step by certain specific membrane proteins, such as V-ATPase, that progressively acidifies the intravesicular milieu [3]. During the process of maturation, the pH values of the vesicles decrease from the extracellular environment (7.4) to early endosomes (6.1–6.8), late endosomes (4.8–6.0) and lysosomes (4.5) [4]. Most importantly, protein-based therapeutics that exert their effect in the cytosol must escape from the endosomes to successfully reach their intracellular targets and exhibit their mechanism of action [5]. Failure to escape the endo/lysosomes leads to the entrapment and subsequent degradation of the protein in the lysosomes [6].

Some bacterial toxins present diverse mechanisms to escape from endosomes and trigger their highly cytotoxic effects. Diphtheria toxin from *Corynebacterium diphtheria* is internalized by clathrin-mediated endocytosis and entrapped first in early endosomes and, subsequently, in late endosomes [7]. It is in these late vesicles where the toxin is able to translocate through the endosomal membrane and reach its target, eukaryotic elongation factor 2 (EF2), in the cytosol [8]. Anthrax toxin from *Bacillus anthracis* is another example of the relevance of the endosomal escape for the successful action of a protein. Anthrax toxin is similarly internalized by clathrin-mediated endocytosis to early and late endosomes, and it will only cause its effect on the mitogen-activated protein kinase kinases (MAPKK) once it has escaped from the late endosomes [9].

Furthermore, plant toxins, such as ribosome-inactivating proteins (RIPs), must also escape from endosomes to reach the cytosol and cleave the 28S rRNA, which will result in apoptosis and cell death [10]. Type II RIPs, such as ricin from *Ricinus communis* L. (comprised of an enzymatic domain and a lectin-binding domain), enter the cell by receptor-mediated endocytosis and follow a complex cell trafficking via retrograde transport and endoplasmic reticulum-associated degradation (ERAD) to finally reach the cytosolic target [11]. In contrast, type I RIPs, such as saporin from *Saponaria officinalis* L. (consisting of only an enzymatic domain and lacking the cell-binding domain), are internalized, probably by receptor-independent endocytosis [12], and accumulate in late endosomes and lysosomes [13], where they get degraded [14]. Type I RIPs can only exhibit cytotoxicity if they can efficiently escape from the endo/lysosomes. In the case of saporin at lower concentrations, this phenomenon may only happen in the presence of certain structurally-specific triterpenoidal saponins, such as SO1861, which naturally occur in the same plant [15]. These triterpenoidal saponins specifically mediate the endo/lysosomal escape of saporin at non-permeabilizing concentrations and the endo/lysosomal membranes remain intact during this process [16].

The successful endo/lysosomal escape in the case of toxins is typically evaluated considering the cytotoxicity of the protein, which will solely appear if the toxin has efficiently crossed the endo/lysosomal membrane to the cytosol. However, for the evaluation of endo/lysosomal escape in the case of other protein-based therapeutics, which are not toxic, the use of a reporter for the endo/lysosomal escape may be required.

In this study, three different reporters for endo/lysosomal escape have been investigated. The reporters were based on the determination of peroxidase activity (horseradish peroxidase), fluorescence (small fluorophore Alexa Fluor 488) and evaluation of cytotoxicity (ricin A-chain). The naturally occurring phenomenon of endo/lysosomal escape enhancement of saporin in combination with structurally-specific triterpenoidal saponins (SA1641 and SO1861) [13,15] was used as a basis to distinguish between the entrapment of the toxin in the endo/lysosomes (saporin alone) and its endo/lysosomal release (saporin plus triterpenoidal saponin). In case of ricin A-chain as a reporter, cytotoxicity is the readout of the experiment. Therefore, a previously described enzymatically inactive variant of saporin (saporin-KQ) [17] was used as a protein that escapes from endo/lysosomes in the presence of triterpenoidal saponins, but that lacks cytotoxic properties. 

## 2. Results and Discussion

### 2.1. Horseradish Peroxidase As a Reporter

**Figure 1:**
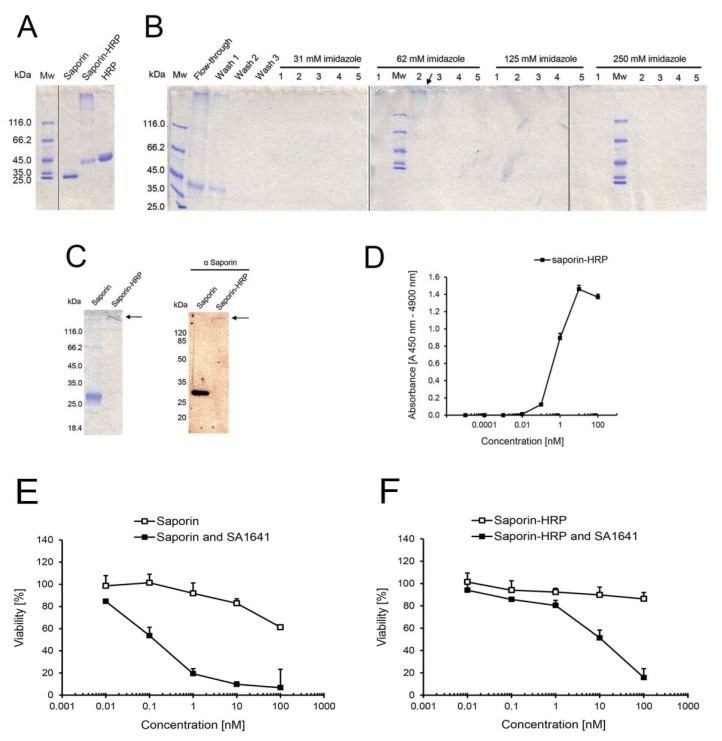
Chemical conjugation, purification and characterization of saporin-HRP. (**A**) Cross-linkage of saporin and HRP. The reaction mixture was directly analysed by SDS-PAGE. Saporin and HRP served as unconjugated controls; (**B**) Purification of saporin-HRP by Ni-NTA chromatography with increasing concentrations of imidazole in the elution buffer. All fractions were assessed by SDS-PAGE. Saporin-HRP was eluted in Fractions 1–3 at 62 mM imidazole (see arrow); (**C**) Validation of the chemical conjugation of saporin and HRP. Saporin-HRP was analysed by SDS-PAGE, and the conjugate was visualized in the gel (see arrow). Saporin-HRP was analysed by Western blot with a primary polyclonal antibody against saporin. Saporin and the conjugate (see arrow) were specifically detected in the membrane; (**D**) Peroxidase activity of saporin-HRP. A directly proportional correlation between absorbance and concentration was observed from 0.1 to 10 nM saporin-HRP. Each data point is the mean ± SD, *n* = 3; Comparison of the cytotoxicity of (**E**) saporin and (**F**) saporin-HRP in the presence of SA1641. ECV-304 cells (4000 cells/well) were treated with saporin or saporin-HRP in a concentration range from 0.01 to 100 nM alone or in combination with SA1641 (final concentration of 5 µg/mL) for 48 h. Cell viability was determined by the MTT assay. Data represents the mean ± SD, *n* = 4.

The first reporter for endo/lysosomal escape that was investigated in this study was horseradish peroxidase (HRP). For this purpose, saporin (recombinantly expressed and purified by Ni- nitrilotriacetic acid (NTA) chromatography; see Figure S1 in the Supplementary) was chemically conjugated to HRP via a covalent linkage, and the reaction mixture was analysed by SDS-PAGE (Figure 1A). A diffused band with high molecular mass was identified by the reaction mixture in the gel. This band in all probability contained conjugates of saporin-HRP with different molecular ratios of saporin to HRP. Furthermore, a band with the same molecular mass as HRP was observed, while no unconjugated saporin was detected in the reaction mixture.

To remove unconjugated HRP, the reaction mixture was purified by Ni-NTA chromatography. The flow-through, wash and elution fractions were analysed by SDS-PAGE (Figure 1B). Unconjugated HRP that did not contain the 6× his-tag was directly eluted in the flow-through and during the washing steps. A certain amount of saporin-HRP was eluted in the flow-through and subsequent washing steps, apparently because the cross-linking reaction of saporin to HRP resulted in the partial masking of the 6× his-tag and prevented the affinity binding of a subset of saporin-HRP to the column. However, saporin-HRP with accessible 6× his-tag bound strongly to the column and was eluted in Fractions 1–3 at 62 mM imidazole (see arrow in Figure 1B). Fractions containing purified saporin-HRP were separately dialyzed and concentrated. The total yield for saporin-HRP was around 1.4 mg of conjugate, and the relative outcome was 14% of the protein input (saporin plus HRP).

Although chemical conjugation was already validated by the specific binding of the conjugate (containing the 6× his-tag in the saporin moiety) to the Ni-NTA agarose and detection of the high molecular mass conjugate after SDS-PAGE (see Figure 1B), validation of the cross-linking reaction was confirmed again by western blot with a primary polyclonal antibody against saporin (Figure 1C). Both saporin (intense band in the unconjugated control) and saporin-HRP (slight band) were specifically detected. Unconjugated saporin was not detected in the conjugate sample.

To determine if the peroxidase activity of HRP had been affected by the chemical conjugation process and to define the sensitivity of the conjugate, peroxidase activity of saporin-HRP was measured at a series of concentrations ranging from 0.00001 to 100 nM (Figure 1D). In the lower concentrations (0.00001 to 0.01 nM), the enzymatic activity of saporin-HRP was not detectible. The first detectable signals in the presence of saporin-HRP appeared at concentrations between 0.01 and 0.1 nM of the conjugate. Then, a linear correlation between the absorbance and concentration of the conjugate was observed in the range from 0.1 to 10 nM saporin-HRP. Finally, for concentrations higher than 10 nM, a saturation of the absorbance signal was recorded.

The *N*-glycosidase activity of saporin-HRP was compared to that of saporin to investigate if the enzymatic activity of the toxic moiety had been influenced by the chemical conjugation process. Saporin-HRP released 46.3 pmol adenine/pmol toxin/h after incubation with herring sperm DNA. Since the adenine release of saporin was calculated to 117.6 pmol adenine/pmol toxin/h, the *N*-glycosidase activity of saporin-HRP was reduced to 39.3%. However, saporin-HRP still presented enzymatic activity and was therefore suitable for further studies.

The effect of triterpenoidal saponins (SA1641) on the enhancement of the endo/lysosomal escape of saporin-HRP was compared to that on saporin on ECV-304 cells. In the case of saporin (Figure 1E), the toxin alone showed cytotoxic effects and caused a reduction of the viability to 61% at a concentration of 100 nM. In combination with SA1641 (5 µg/mL), saporin cytotoxicity was drastically enhanced, causing a reduction of cell viability to 54% at a concentration of 0.1 nM. 

In the case of saporin-HRP (Figure 1F), no cytotoxicity was shown up to a concentration of 100 nM. However, in the presence of SA1641, saporin-HRP exerted cytotoxicity at the two highest concentrations tested: cell viability was reduced to 51% at 10 nM and to 16% at 100 nM. It is important to note that the cytotoxicity enhancement also took place when saporin was chemically coupled to HRP, therefore indicating that the endo/lysosomal escape of saporin in combination with triterpenoidal saponins was coherent, even after coupling to the reporter, and that saporin has a general potential as a molecular drag.

In order to measure the specific augmentation of the endo/lysosomal release of saporin-HRP mediated by triterpenoidal saponins, cells were first treated either with saporin-HRP (100 nM) or with the combination of saporin-HRP and SA1641 (5 µg/mL). After an incubation time of 6 h and cell fractionation, the endo/lysosomal release of the toxin-reporter conjugate was evaluated by measuring peroxidase activity in the cytosolic fraction and in the lysosomal fraction. The ratio of the peroxidase activity in the cytosolic fraction and the peroxidase activity in the lysosomal fraction (CF/LF) is proportional to the relative amount of saporin-HRP released to the cytosol. 

Unfortunately, the CF/LF value mirrored contradictory results in repeated experiments. Peroxidase activity detected in the cytosolic fractions was very close to the detection limit (A_450–490 nm_ = 0.018–0.030), and therefore, small variations in the absorbance values greatly influenced the CF/LF ratio. In one example, the CF/LF value of the cells treated with SA1641 (0.082) was higher than that of the cells treated without the triterpenoidal saponin (0.072). However, in another example, the CF/LF value of the cells treated with SA1641 (0.107) was lower than that of the cells treated without the triterpenoidal saponin (0.151). In short, the measurements close to the detection limit resulted in a bad reproducibility and prevented the precise calculation of ratios representing the endo/lysosomal escape of saporin-HRP in the presence or absence of triterpenoidal saponins. 

**Figure 2:**
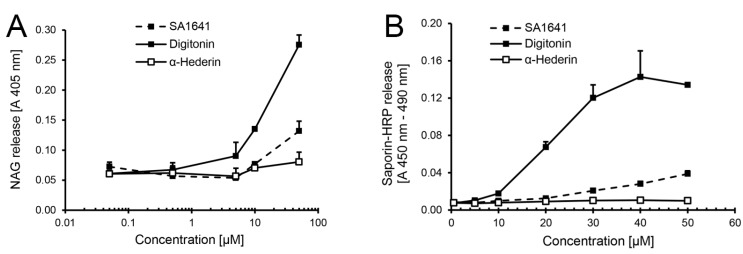
HRP as a reporter for endo/lysosomal escape in isolated organelles. Lysosomes were isolated by cell fractionation form ECV-304 cells (3 × 10^7^ cells). (**A**) First, the permeabilizing effects of SA1641 on the lysosomal membranes were evaluated by the β**-***N*-acetylglucosaminidase (NAG) release assay.The permeabilizing effects of digitonin (highly lytic saponin) and α-hederin (non-lytic saponin) were simultaneously determined as controls; (**B**) Thereafter, the endo/lysosomal escape enhancement of saporin-HRP observed in the cytotoxicity assay in Figure 1F was tried, to monitor in isolated lysosomes loaded with saporin-HRP (from cells previously treated with the conjugate at 100 nM and 37 °C for 6 h) by determination of the peroxidase activity. Saponin concentrations are shown in µM to allow a better comparison. Each data point represents the mean ± SD, *n* = 3.

In an alternative approach, the endo/lysosomal escape of saporin-HRP was evaluated in isolated organelles preloaded with the conjugate. To determine the highest non-permeabilizing concentration of triterpenoidal saponins, short-term permeabilizing effects of SA1641 on lysosomal membranes from isolated organelles were measured using the lysosomal enzyme β**-***N*-acetylglucosaminidase (NAG) release assay (Figure 2A). In the case of digitonin (positive control, highly lytic saponin isolated from *Digitalis purpurea* L.), slight membrane permeabilizing effects on the lysosomal membrane were first observed at a concentration of 5 µM (6.15 µg/mL). The permeabilizing effects of digitonin progressively increased as the concentration was augmented to 50 µM (61.47 µg/mL). In the case of α-hederin (negative control, non-lytic saponin isolated from *Hedera helix* L.), no membrane permeabilizing effects were observed on the lysosomal membrane. Even at the highest concentration tested, only a very slight release of NAG was measured. SA1641 had substantial membrane permeabilizing effects at a concentration of 50 µM (82.05 µg/mL). However, at lower concentrations, SA1641 did not present any permeabilizing effects on lysosomal membranes, and the absorbance values were comparable to those of α-hederin.

In the case of a specific augmentation of the endo/lysosomal release of saporin-HRP in the presence of SA1641, higher amounts of the conjugate are expected to be released at low non-membrane permeabilizing concentrations of SA1641 (lower than 50 µM or 82.05 µg/mL). In such a case, saporin-HRP will be released from the lysosomes, due to a specific interaction with SA1641.

Nevertheless, no such specific release of saporin-HRP was observed in the presence of SA1641 (Figure 2B). Although cytotoxicity enhancement is already observed at 5 µg/mL of SA1641 (Figure 1F), no specific endo/lysosomal release of saporin-HRP was observed at this concentration. The endo/lysosomal release of saporin-HRP increased slightly as the concentration was augmented to 30, 40 and 50 µM (49.23, 64.64 and 82.05 µg/mL, respectively), most probably attributed to unspecific membrane permeabilizing effects. In the case of digitonin, the high endo/lysosomal release of saporin-HRP was shown at 30, 40 and 50 µM (36.88, 49.17 and 61.47 µg/mL, respectively), due to disruption of lysosomal membranes. In the case of α-hederin, no endo/lysosomal release of saporin-HRP was observed at all the concentrations tested. Regrettably, the reporter assay for endo/lysosomal escape using HRP is not suitable to quantify the endo/lysosomal release of protein-based therapeutics.

It is known from previous studies that the release of diphtheria toxin’s catalytic (C) domain from endosomes into the cytosol requires a host cell cytosolic translocation factor complex, which consists of the chaperone heat shock protein 90 (Hsp 90) and thioredoxin reductase [18]. Furthermore, host cell Hsp 90 is also essential for the translocation of *Clostridium botulinum* C2 toxin from endosomes into the cytosol [19] and for the transfer of the cholera toxin A1 subunit from the endoplasmic reticulum to the cytosol [20]. Similarly, since a certain amount of saporin-HRP must definitively escape from the endo/lysosomes in the presence of triterpenoidal saponins, because cytotoxicity was observed (see the cytotoxicity enhancement at 3.05 µM or 5 µg/mL of SA1641 in Figure 1F), a possible explanation for the lack of saporin-HRP release from the isolated organelles (see saporin-HRP release at 5 µM or 8.21 µg/mL SA1641 in Figure 2B) is the necessity of a certain cytosolic machinery to efficiently mediate the specific endo/lysosomal release of saporin (or saporin conjugates, such as saporin-HRP) in the presence of triterpenoidal saponins. An alternative hypothesis is that the amount of saporin-HRP that escapes from the isolated endo/lysosomes is so low that its measurement remains under the limit of detection, but it is high enough to cause cell death.

### 2.2. Alexa Fluor 488 As a Reporter

**Figure 3:**
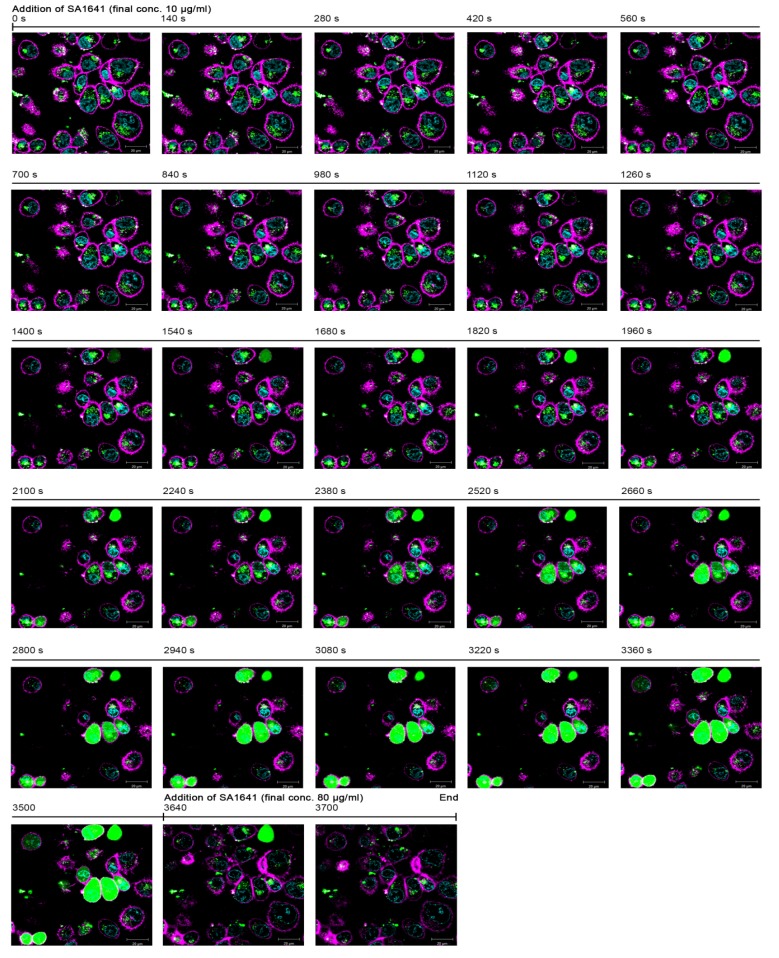
Alexa Fluor 488 as a reporter for endo/lysosomal escape in live cell imaging. The report of the naturally occurring phenomenon of the endo/lysosomal escape enhancement of saporin (in this case ^Alexa^saporin) was assayed by constant measurement of fluorescence. ECV-304 cells were incubated with 1000 nM ^Alexa^saporin at 37 °C for 3 h. Afterwards, most of the internalized toxin accumulated in intracellular vesicles. ^Alexa^saporin is visualized in green fluorescence, cell membranes in magenta and cell nuclei in cyan. The addition of 10 µg/mL SA1641 (non-cytotoxic concentration) was defined as t = 0 s. Green colouring of the cells reflects the release of ^Alexa^saporin (from t = 1400 s to t = 3500 s). To finish the experiment, SA1641 was added (t = 3640 s) to a final concentration of 80 µg/mL (cytotoxic concentration), which results in total cell membrane disruption (from t = 3640 s to t = 3700 s).

The second reporter that was investigated was Alexa Fluor 488. Recombinantly expressed saporin was labelled with Alexa Fluor 488, and the conjugate (^Alexa^saporin) was administered to cells in the presence of SA1641 to visualize the endo/lysosomal escape of saporin by live cell imaging (Figure 3).

After the incubation of cells with ^Alexa^saporin for 3 h, only the accumulation of the labelled protein (emitting green fluorescence) was observed inside the cell, but no endo/lysosomal escape was detected. Cells treated with ^Alexa^saporin under the same conditions for 6 h indicated that the toxin accumulates in acidic organelles, namely late endosomes and lysosomes, but is not released to the cytosol [13]. Furthermore, in the present experiment, ^Alexa^saporin did presumably not have any cytotoxic effects in the short incubation time of 3 h, because the morphology of the cells was intact. At that moment (t = 0 s), triterpenoidal SA1641 was added to the cells to a final concentration of 10 µg/mL, and initially, no changes were observed. However, after 1400 s of addition of SA1641, a diffusion of green fluorescence started to become visible in a cell at the top right of the fluorescence microscopy picture. This diffusion was limited to the intracellular space and was associated with the endo/lysosomal escape of ^Alexa^saporin. 

Within the next 300 s (from t = 1400 s to t = 1680 s), the intensity of fluorescence in the cell increased rapidly and reached a saturation point, indicating a complete release of ^Alexa^saporin into the cytosol. Furthermore, after 2100 s, fluorescence diffusion appeared in two more cells: one at the top centre and another one at the bottom left of the fluorescence image. After 2800 s, the fluorescence diffusion was observed in seven cells, and after 3500 s, a total of nine cells exhibited diffused fluorescence in the cytosol. It is remarkable that the diffusion of the fluorescence occurred only in the intracellular space, but did not cross the cell membrane, indicating that SA1641 did not cause unspecific cell membrane permeabilization, but specifically mediated the endo/lysosomal escape of ^Alexa^saporin. Only after the addition of SA1641 at a high concentration (80 µg/mL), cell membranes were unspecifically permeabilized, and fluorescence diffusion disappeared quickly, due to the leakage of ^Alexa^saporin from the cytosol to the extracellular space. A video comprising the whole sequence of images is available in the Supplementary (Video S1). The single cell analysis derived from the fluorescence images in the Video S1, but not the fluorescence images themselves, was previously published [13].

**Figure 4:**
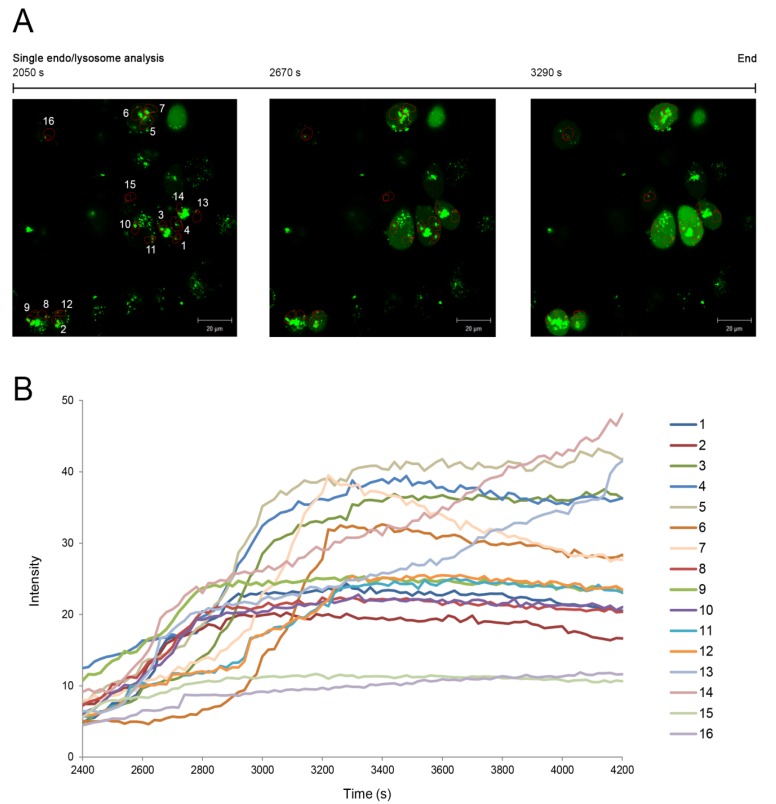
Single endo/lysosome analysis. The endo/lysosomal escape of ^Alexa^saporin in the presence of SA1641 was monitored in the case of single endo/lysosomes. (**A**) ECV-304 cells were incubated with ^Alexa^saporin at 1000 nM and 37 °C for 3 h, washed, and then, 10 µg/mL SA1641 were added to cells as indicated in Figure 3. ^Alexa^Saporin is visualized in green fluorescence. A total of 16 single endo/lysosomes was selected and constantly monitored from t = 2050 s to t = 3290 s. For each endo/lysosome, a region of interest (numbered 1–16 in the fluorescence picture) comprised of the surroundings of the endo/lysosomes, but excluding the vesicle itself, was defined; (**B**) ECV-304 cells were continuously monitored for 7200 s after the addition of 10 µg/mL of SA1641. Single endo/lysosome analysis is presented as a relation of time (from t = 2400 s to t = 4200 s) *vs.* the fluorescence intensity of the released ^Alexa^saporin. Each curve represents the fluorescence intensity increase in each region of interest, corresponding to the amount of ^Alexa^saporin that escapes from each single endo/lysosome.

In addition, the endo/lysosomal escape was analysed in this study for 16 single endo/lysosomes identified in the fluorescence images from 2050 s to 3290 s at a final concentration of 10 µg/mL (Figure 4A). Regions of interest were defined for each endo/lysosome, and the amount of green fluorescence diffusion that corresponds to the released amount of ^Alexa^saporin was individually evaluated in the case of each endo/lysosome. Furthermore, in a second experiment, where cells were monitored for 7200 s after the addition of SA1641, the fluorescence intensity of ^Alexa^saporin released from individual endo/lysosomes was plotted against the time (from t = 2400 s to t = 4200 s) (Figure 4B). A curve for each of the 16 endo/lysosomes was obtained and every curve presented a particular behaviour, showing the differences in the release rate of ^Alexa^saporin from one endo/lysosome to another.

While Figure 1E demonstrates that saporin at 1000 nM in combination with SA1641 produces close to 100% total cell death, in Figure 3, only a portion of cells treated by saporin at the same concentration in the presence of SA1641 displays the cytosolic fluorescence indicative of the toxin release to the cytosol. This discrepancy may be related to the timing of the experiments and also to the detection limits of the techniques. 

Firstly, regarding the timing of the experiments, results presented in Figure 1E refer to cells treated for 48 h, whereas results in Figure 3 represent cells treated first with saporin alone for 3 h and thereafter with the combination of saporin and SA1641 for 2 h more. It is therefore expected that a major number of cells will be affected in the first case, since cells have been in contact with both compounds for a longer period of time. 

Secondly, concerning the detection limits, the sensitivity of the cytotoxicity assay reaches much lower concentrations than that of confocal microscopy. In Figure 1E, cytotoxicity is observed down to a concentration of 0.1 nM. However, the fluorescence diffusion of toxins after endo/lysosomal escape is clearly observed only at 1000 nM (see Figure 3). When an immunotoxin consisting of saporin chemically coupled to the therapeutic antibody, Trastuzumab (Herceptin^®^), was labelled with Alexa Fluor 488, its endo/lysosomal escape was also observed at a toxin concentration of 100 nM, but the diffusion of fluorescence to the cytosol was not as clear as in the case of ^Alexa^saporin at 1000 nM [16]. To sum up, the effects of saporin are detected in a major number of cells in the cytotoxicity assay, also probably due to the higher sensitivity of the method.

Alexa Fluor 488 was previously used as a reporter for endo/lysosomal escape of saporin in the presence of triterpenoidal saponins. First, cells were treated with ^Alexa^saporin in combination with SA1641 [13]. Later, ^Alexa^saporin was administered to cells in the presence of SO1861 [21]. In both cases, the endo/lysosomal escape of the toxin was observed at a toxin concentration of 1000 nM. Based on the previous experiments, the limit of detection with the reporter system for endo/lysosomal escape based on Alexa Fluor 488 is approximated to 100–1000 nM. A novelty of the present study is the single endo/lysosome analysis of cells incubated with ^Alexa^saporin (also at 1000 nM). To summarize the results of this section, a concentration of 1000 nM was a suitable concentration for single-endo/lysosome analysis and for the usage of Alexa Fluor 488 as a reporter for the endo/lysosomal escape of protein-based therapeutics.

### 2.3. Ricin A-Chain As a Reporter

The third reporter for the endo/lysosomal escape considered in this investigation was the ricin A-chain (RTA). In order to study the usage of RTA as a reporter, it was chemically conjugated to an enzymatically inactive variant of saporin (saporin-KQ), previously reported in the literature [17]. Saporin-KQ was cloned into an expression vector, and the presence of the insert was confirmed by DNA sequencing. Then, saporin-KQ was heterologously expressed and purified by Ni-NTA chromatography (Figure S2A in the Supplementary). The identity of saporin-KQ after recombinant expression and purification was substantiated by Western blot with a primary polyclonal antibody against saporin (Figure S2B, Supplementary).

**Figure 5:**
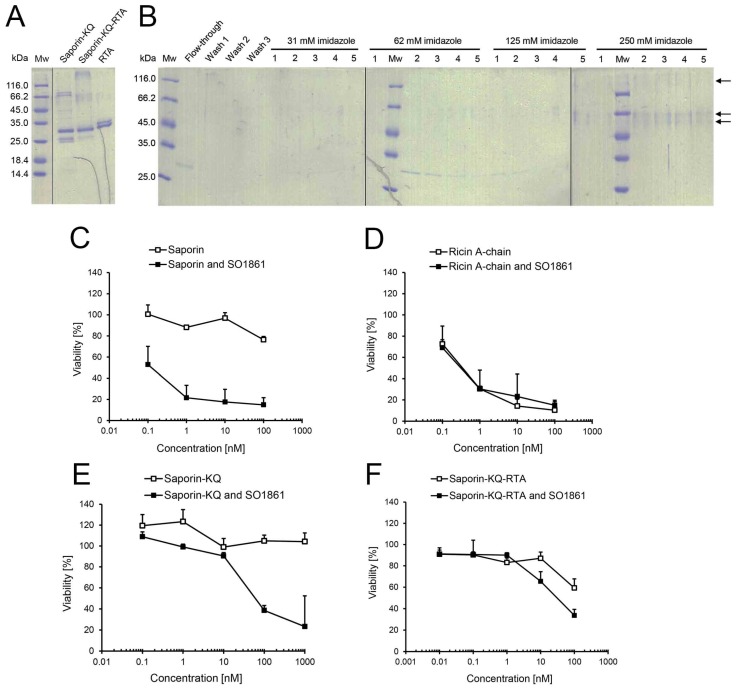
Ricin A-chain (RTA) as a reporter for endo/lysosomal escape. The report of the endo/lysosomal escape enhancement of saporin-KQ (an enzymatically inactive saporin in order to avoid interferences in the readout of the experiment) was attempted by measuring the cytotoxic activity of RTA. (**A**) Saporin-KQ and RTA were chemically cross-linked. The reaction mixture was analysed by SDS-PAGE, and both unconjugated saporin-KQ and RTA were included as controls; (**B**) Saporin-KQ-RTA was purified by Ni-NTA chromatography. Proteins were eluted with increasing concentrations of imidazole (31, 62, 125 and 250 mM), and an SDS-PAGE analysis was effectuated for the fractions.Saporin-KQ-RTA was eluted in Fractions 5 at 125 mM and 1–5 at 250 mM imidazole (see arrows); Cytotoxicity of (**C**) saporin; (**D**) RTA; (**E**) saporin-KQ; and (**F**) saporin-KQ-RTA in combination with SO1861. ECV-304 (4000 cells/well) were treated with saporin, RTA, saporin-KQ or saporin-KQ-RTA in a concentration range from 0.01 to 1000 nM in the presence or absence of SO1861 (final concentration of 1–2 µg/mL) for 48 h. The viability of cells was evaluated by the MTT assay. Data is the mean ± SD, *n* = 4.

After chemical conjugation of saporin-KQ and RTA, a first diffused band with an approximate molecular mass of 60 kDa and a second diffused band with high molecular mass were observed in the reaction mixture by SDS-PAGE (Figure 5A). The first band contained most likely one single saporin-KQ coupled to a single molecule of RTA. In contrast, the second band may probably contain conjugates of saporin-KQ-RTA with different molecular ratios of saporin-KQ conjugated to RTA. Furthermore, a band of approximately 30 kDa was detected, as well, in the reaction mixture, but since saporin-KQ and RTA have a very similar molecular mass, the presence of either one or the other protein cannot be distinguished.

To purify the saporin-KQ-RTA conjugates from the unconjugated saporin-KQ and unconjugated RTA, Ni-NTA chromatography was carried out, and all the fractions were analysed by SDS-PAGE (Figure 5B). On the one hand, unconjugated RTA was directly eluted in the flow-through, as it lacks the 6× his-tag. On the other hand, unconjugated saporin-KQ (presenting the 6× his-tag) was eluted in Fractions 1–5 at 62 mM and 1–2 at 125 mM imidazole. In the case of saporin-KQ-RTA (also presenting the 6× his-tag), the conjugate was eluted in Fractions 5 at 125 mM and 1–5 at 250 mM imidazole. All fractions that contained the purified conjugate were pooled together, desalted and concentrated. The final amount of saporin-KQ-RTA was 0.26 mg of conjugate, and the relative yield of the chemical conjugation and subsequent purification was 18% of protein input (saporin-KQ plus RTA).

In order to check that saporin-KQ was enzymatically inactive, the *N*-glycosidase activity of saporin was compared to that of saporin-KQ. In the case of saporin, the release of adenine after incubation with herring sperm DNA was 117.6 pmol adenine/pmol toxin/h (see Section 2.1). However, in the case of saporin-KQ, no adenine release was observed after the incubation of the inactive mutant with the substrate. In brief, the enzymatic inactivity of saporin-KQ was confirmed.

The effect of triterpenoidal saponins (SO1861 at 1–2 µg/mL) on the enhancement of the endo/lysosomal escape of saporin-KQ was compared to that on saporin and RTA. Saporin alone presented cytotoxicity at a concentration of 100 nM and reduced the viability to 76% (Figure 5C). In combination with SO1861, the cytotoxicity of saporin was tremendously augmented, and cell viability was reduced to 53% at a concentration of 0.1 nM. These results are comparable to those obtained for the combination of saporin and SA1641 in Figure 1E. Both triterpenoidal saponins (SA1641 and SO1861) have been reported for the specific enhancement of the endo/lysosomal escape of saporin (SA1641 in [13] and SO1861 in [21]), and in both cases, this phenomenon has been elucidated by cytotoxicity assays, as well as by confocal microscopy. The endo/lysosomal escape of saporin mediated by both triterpenoidal saponins is consequently based on the same molecular mechanism, and this allows the comparison between the different reporters presented in this study.

In the case of RTA (Figure 5D), the toxin alone was already very cytotoxic at 1 and 10 nM (cell viability of 31% and 14%, respectively). RTA at a final concentration of 0.1 nM was also cytotoxic, and cell viability was reduced to 73%. When RTA was applied in combination with SO1861, the toxin presented very similar cytotoxic effects as before with no significant differences. The drastic cytotoxicity augmentation of saporin in the presence of triterpenoidal saponins was not observed for RTA. These results are in accordance with the literature that indicates that SO1861 specifically modulates the intracellular trafficking of saporin [13] and that RTA and saporin follow different intracellular routes to enter the cytosol [22].

The cytotoxicity of saporin-KQ was also evaluated alone or in combination with SO1861 to assure that the enzymatically inactive saporin was non-cytotoxic (Figure 5E). According to its mutation, which turns saporin-KQ into an enzymatically inactive protein, saporin-KQ did not present any cytotoxicity up to 1000 nM. Nevertheless, in the presence of SO1861, saporin-KQ caused the inhibition of cell growth at the two highest concentrations tested (presumably due to known mechanisms unrelated to enzymatic activity; see below), and cell viability was reduced to 39% (100 nM) and 23% (1000 nM), indicating an endo/lysosomal escape enhancement of saporin-KQ in the presence of triterpenoidal saponins. As cells cannot be killed by the enzymatic activity of saporin-KQ (inactive mutant), it is hypothesized that saporin-KQ induces caspase-dependent apoptosis via the mitochondrial or intrinsic pathway, independently of translation inhibition [23].

Finally, the cytotoxicity of saporin-KQ-RTA was evaluated in the presence of SO1861 to find out whether the endo/lysosomal escape of saporin-KQ was efficiently reported by RTA (Figure 5F). When saporin-KQ-RTA was tested alone, it was cytotoxic only at the highest concentration tested (100 nM), reducing cell viability to 59%. In contrast, in the presence of SO1861, saporin-KQ-RTA was cytotoxic already at a concentration of 10 nM lowering cell viability to 65% and also at 100 nM, decreasing cell viability to 34%. Remarkably, the cytotoxicity of saporin-KQ-RTA was enhanced in the presence of triterpenoidal saponins. Considering that the cytotoxicity of the reporter RTA is not enhanced by SO1861 (see Figure 5D) and that the cytotoxic effects of saporin-KQ in the presence of SO1861 only appear with concentrations higher than 100 nM (see also Figure 5E), the cytotoxicity enhancement of saporin-KQ-RTA at 10 nM mediated by SO1861 can only be explained by the endo/lysosomal escape enhancement of the saporin-KQ moiety and the cytotoxicity of the RTA reporter once the conjugate has reached the cytosol. Thus, the reporter assay based on RTA was able to detect the endo/lysosomal escape with a sensitivity down to 10 nM.

Theoretically, since it has been reported that the presence of a single molecule of ricin in the cytosol is enough to trigger apoptosis and thus kill a cell [24], the strategy of using RTA as a reporter would allow the achievement of sensitivities even lower than 10 nM. From the present investigations, it can be certainly concluded that RTA is only an appropriate reporter for protein therapeutics affected by structurally-specific triterpenoidal saponins. However, we hypothesize that RTA could be further used as an endo/lysosomal escape reporter for protein therapeutics that are affected by compounds (endo/lysosomal escape enhancers) that do not cause any effects on RTA and for proteins therapeutics that achieve the endo/lysosomal escape in concentrations lower than the cytotoxicity of RTA alone (<0.1 nM, see Figure 5D). This methodology confers the possibility of detecting small quantitative differences, which only take place at very low concentrations, in the endo/lysosomal escape of a protein-based therapeutic under different conditions.

## 3. Experimental Section

### 3.1. Molecular Cloning of Saporin and Saporin-KQ

The plasmid codifying for saporin (6× his-tagged-saporin-pET11d) was cloned in previous studies [25]. The DNA for saporin-KQ previously reported in the literature [17] was synthesized and cloned into the pEN08H vector (6× his-tagged-saporin-KQ-pEN08H) by Entelechon (Regensburg, Germany). To insert the DNA for saporin-KQ in the expression vector, pET11d (Novabiochem, Schwalbach, Germany), the 6× his-tagged-saporin-KQ-pEN08H and 6× his-tagged-dianthin-30- epidermal growth factor-pET11d [26] were digested by *Nco*I and *Eco*RI-HF (New England Biolabs, Ipswich, MA, USA). The digested DNA for saporin-KQ and the digested vector pET11d were separated from the other digestion products by agarose gel electrophoresis, and appropriate DNA bands were extracted with the Zymoclean Gel DNA Recovery Kit (Zymo Research, Irvine, CA, USA). The concentration of the extracted digested products was determined by the Nanodrop ND-1000 Spectrophotometer (Peqlab, Erlangen, Germany). Insert and vector (molar ratio of 3:1) were ligated by T4 DNA ligase (New England Biolabs, Ipswich, MA, USA). The resulting plasmid 6× his-tagged-saporin-KQ-pET11d was transformed and propagated in *Escherichia coli* Library Efficiency DH5α Competent Cells (Life Technologies, Carlsbad, CA, USA). The plasmid was extracted by the Zyppy Plasmid Miniprep Kit (Zymo Research, Irvine, CA, USA). The DNA sequence for saporin-KQ was sequenced by the method of BigDye Terminator Sequencing (Applied Biosystems, Carlsbad, CA, USA) using a forward primer binding at the T7 promoter, at the 5’-end of the insert (5’-TAATACGACTCACTATAG-3’), and a reverse primer binding at the pET11d vector, at the 3’-end of the insert (5’-CCTGACGTCTAAGAAACC-3’). Samples were sequenced with the ABI PRISM 310 Genetic Analyser (Advanced Biolab Service, München, Germany).

### 3.2. Heterologous Expression of Recombinant Proteins

The plasmids coding for saporin (6× his-tagged-saporin-pET11d) or saporin-KQ (6× his-tagged-saporin-KQ-pET11d) were transformed into *Escherichia coli* Rosetta 2(DE3) pLysS Competent Cells (Novagen, San Diego, CA, USA). The proteins were expressed as described elsewhere [27]. In short, the bacteria comprising the corresponding plasmid were grown up to a culture of 2 L, until an optical density A_600nm _= 0.9, and protein expression was induced by the addition of isopropyl β-d-1-thiogalactopyranoside (IPTG) (final concentration of 1 mM). Protein expression was allowed for 3 h at 37 °C. Thereafter, the bacterial suspension was centrifuged, pellets were re-suspended in phosphate-buffered saline (PBS) and stored at –20 °C until further use.

### 3.3. Chemical Conjugation of Reporters to Saporin

Saporin (see Section 3.4 for its purification) was chemically conjugated to the reporter HRP (Serva Electrophoresis, Heidelberg, Germany) via modification of the *N*-glycans of HRP by sodium periodate (NaIO_4_) oxidation and sodium borohydride (NaBH_4_) reduction. Freshly prepared 0.1 M NaIO_4 _(185.05 µL) was added to 7.30 mg HRP (previously dissolved in 2 mL water), and the solution was stirred for 20 min at room temperature protecting it from light. The modified HRP was dialyzed overnight at 4 °C against 8 L of 1 mM sodium acetate buffer (pH 4.4). In parallel, 2 mL saporin (1.235 mg/mL) was buffered with 200 µL sodium hydrogen carbonate buffer (0.2 M, pH 9.5). The pH value of the dialyzed modified HRP was adjusted to 9.0–9.5 by adding 80 µL sodium hydrogen carbonate buffer, and it was immediately mixed with the saporin solution (molar ratio of 2:1 for HRP:saporin). The mixture was stirred for 2 h at room temperature. After the incubation time, 100 µL of freshly prepared NaBH_4_ (4 mg/mL) was added to the mixture, and it was further stirred for 2 h at 4 °C. The protein solution was dialyzed overnight against 2 L PBS. The saporin-HRP solution was further purified by Ni-NTA affinity chromatography.

Saporin was conjugated to the reporter Alexa Fluor 488 carboxylic acid, 2,3,5,6-tetrafluorophenyl ester (Alexa Fluor 488 5-TFP) (Molecular Probes, Eugene, USA), by adding 100 μL sodium hydrogen carbonate buffer (1 M, pH 9.0) and 100 μL of Alexa Fluor 488 5-TFP (5 mg/mL in DMSO) to 750 µL of saporin (1.2 mg/mL in PBS). The samples were cross-linked for 1 h at room temperature and then dialyzed overnight at 4 °C against 2 L PBS to purify the conjugate ^Alexa^saporin from unconjugated Alexa Fluor 488 5-TFP.

Saporin-KQ (details for purification in Section 3.4) was chemically conjugated to the reporter RTA (buffered aqueous glycerol solution, Sigma-Aldrich, Steinheim, Germany) by the same chemical procedure described before in the case of the saporin-HRP conjugate. Freshly prepared 0.1 M NaIO_4 _(185.05 µL) was added to 2.30 mg RTA (previously dialyzed in water and diluted to a volume of 2.85 mL). After the overnight dialysis of the modified RTA against sodium acetate buffer, 400 µL sodium hydrogen carbonate buffer was added to 2 mL saporin-KQ (0.250 mg/mL). Subsequently, 400 µL sodium hydrogen carbonate buffer was added to the RTA solution, and it was immediately mixed with the saporin-KQ solution (molar ratio of 2:1 for RTA:saporin-KQ). The chemical conjugation was finished by the addition of freshly prepared NaBH_4_ (100 µL, 4 mg/mL), the incubation of the mixture and overnight dialysis in PBS. Saporin-KQ-RTA solution was also purified by Ni-NTA affinity chromatography.

### 3.4. Purification of Proteins and Conjugates

After heterologous expression of recombinant proteins, bacterial suspensions containing saporin or saporin-KQ were thawed on ice, and bacteria lysis was achieved by sonication (G. Heinemann, Schwäbisch Gmünd, Germany). Bacteria debris was separated by centrifugation at 15,800 × *g* and 4 °C for 30 min, and after the addition of imidazole (final concentration of 20 mM), recombinant proteins were purified from the supernatants by Ni-nitrilotriacetic acid (NTA) agarose affinity chromatography (Protino Ni-NTA agarose, Macherey-Nagel, Düren, Germany). Ni-NTA agarose (1.5–2.0 mL) was added to the supernatants or the conjugate solutions (saporin-HRP and saporin-KQ-RTA, see Section 3.3) and the 6× his-tagged proteins bound to the matrix under continuous shaking during 30 min at 4 °C. The affinity chromatography was initiated after pouring the matrix with the bound proteins into a 20-mL column (Econo-Pac chromatography columns, Bio-Rad, Hercules, CA, USA). The column was washed three times with 10 mL wash buffer (50 mM NaH_2_PO_4_, 300 mM NaCl, 20 mM imidazole), and the 6× his-tagged proteins were eluted with increasing concentrations of imidazole (31, 62, 125 and 250 mM) in wash buffer. In total, 5 fractions of 2 mL per each concentration were collected. Flow-through, washes and each of the fractions were analysed by SDS-PAGE (12% (*w/v*) gel). Fractions containing the protein of interest were pooled and dialyzed overnight at 4 °C against 2 L PBS. The desalted protein was concentrated via centrifugation with an Amicon Ultra-15 10,000 NMWL (Merck Millipore, Carrigtwohill, Ireland), and the protein concentration was finally determined by a bicinchoninic acid (BCA) assay (Pierce/Thermo Scientific, Waltham, MA, USA).

### 3.5. Identity Validation of Recombinant Proteins and Conjugates

To validate the identity of recombinantly expressed saporin and saporin-KQ, the proteins were analysed by western blot with a primary self-raised rabbit polyclonal antibody against saporin (1:300 diluted). Polyclonal goat anti-rabbit immunoglobulins/horseradish peroxidase (1:10,000 diluted) (P0448, Dako Cytomation, Hamburg, Germany) was used as secondary antibody, and its presence was detected by the enhanced chemiluminescence reaction. After exposure, the photographic paper (Amersham Hyperfilm ECL, GE Healthcare, Uppsala, Sweden) was developed by an Optimax X-Ray Film Processor (Protec Medizintechnik, Oberstenfeld, Germany). The validation of the chemical conjugation of saporin and HRP was effectuated following the same methodology.

### 3.6. Characterization of Peroxidase and N-Glycosidase Activity

The peroxidase activity of saporin-HRP was measured by adding 100 µL of the conjugate (concentrations from 0.00001 to 100 nM) to 100 µL of reagent (80 mM citric acid, 0.4 mg/mL 3,3’,5,5’-tetramethylbenzidine, 0.2 µg/mL H_2_O_2_, pH 3.95). The mixture was incubated for 15 min at room temperature. The reaction was stopped by the addition of 50 µL H_2_SO_4_ (3.3 M), and the absorbance of the mixture was measured at 450 nm (reference at 490 nm) by a SpectraMax 340PC Absorbance Microplate Reader (Molecular Devices, Sunnyvale, CA, USA).

The procedure for the determination of peroxidase activity was modified to record the peroxidase activity of saporin-HRP in cellular fractions (see Section 3.8). Either 50 µL of the (1) cytosolic fraction or (2) lysosomal fraction were added to 20 µL citrate buffer (80 mM citric acid, pH 3.95). After incubation of the fractions for 5 min, the precipitate was discarded by centrifugation at 16,100 × *g* for 2 min. Then, 50 µL of the reagent were added to the supernatant. The mixture was incubated for 1 h at room temperature, and the reaction was stopped by adding 50 µL H_2_SO_4_ (3.3 M).

To measure the activity of saporin-HRP released from isolated lysosomes, the protein solution (20 µL of supernatant after the last centrifugation step; see Section 3.8) was mixed first with 30 µL translocation buffer (20 mM HEPES, 10 mM MgSO_4_ and 0.1 M KCl, pH 7.0). Thereafter, 50 µL of reagent were added, and the mixture was incubated for 1 h at room temperature. The reaction was stopped by adding 25 µL H_2_SO_4_ (3.3 M).

The *N*-glycosidase activity of proteins was determined by the cleavage and release of adenine residues from herring sperm DNA [26,28]. First, a standard curve was prepared with adenine by measuring the absorbance at 260 nm (reference at 300 nm) for concentrations of 10, 20, 40, 80, 160 and 320 pmol/µL adenine. Absorbance was measured by the Nanodrop ND-1000 Spectrophotometer.

To determine the *N*-glycosidase activity of saporin-HRP and saporin-KQ, 10 µL herring sperm DNA (100 µg) (Invitrogen, Darmstadt, Germany) was added to 30 pmol saporin (control), saporin-HRP or saporin-KQ and acetate buffer (50 mM CH_3_COONa, 100 mM KCl, pH 5.0) up to 50 µL. The mixture was incubated under continuous shaking at 50 °C for 1 h. Thereafter, the mixture was transferred to a filtration device (MWCO 3000 Da) (Millipore, Billerica, MA, USA) and centrifuged at 4 °C and 5000 × *g* for 45 min. The absorbance of the filtrate was measured at 260 nm (reference at 300 nm).

### 3.7. Cytotoxicity Assays

ECV-304 cells (human urinary bladder carcinoma cell line) (ACC 310, Deutsche Sammlung von Mikroorganismen und Zellkulturen, Braunschweig, Germany) were cultured in Dulbecco’s modified Eagle’s medium (DMEM) (PAA Laboratories, Pasching, Austria) supplemented with 10% foetal bovine serum (FBS) (BioChrom KG, Berlin, Germany) and 1% penicillin/streptomycin (PS) (Gibco/Invitrogen, Karlsruhe, Germany).

The cytotoxicity of the combination of SA1641 (purified by HPLC as indicated in [29]) with saporin or saporin-HRP was evaluated by first adding 100 µL/well of DMEM supplemented with 10% FBS and 1% PS containing 4000 ECV-304 cells into a 96-well plate. Cells were allowed to grow for 24 h, and thereafter, the medium was removed. Then, 180 µL/well fresh medium or medium containing SA1641 (final concentration of 5 µg/mL) and 20 µL/well PBS containing either saporin or saporin-HRP (final concentrations from 0.01 to 100 nM) were added, and cells were incubated for a further 48 h. Cells treated with 180 µL/well fresh medium and only 20 µL/well PBS served as the control. To determine the cytotoxicity of the combination of SO1861 (isolated by HPLC as described in [15]) with saporin, RTA, saporin-KQ or saporin-KQ-RTA, 180 µL/well medium containing SO1861 (final concentration of 1–2 µg/mL) and 20 µL/well PBS containing saporin, RTA, saporin-KQ or saporin-KQ-RTA (final concentrations from 0.01 to 1000 nM) were added.

To measure the degree of cell proliferation after the treatment of cells under the different conditions, the final viable cell number was determined by the 3-(4,5-dimethylthiazol-2-yl)-2,5-diphenyltetrazolium bromide (MTT) assay. A solution of MTT (5 mg/mL, 30 µL/well) was pipetted directly to the media. The plate was incubated at 37 °C for 2 h, and thereafter, the media containing MTT was removed. A formazan solubilizer (50 µL/well) consisting of 82% (*v/v*) isopropanol (pure), 10% (*v/v*) of SDS solution (10% (*w/v*)) and 8% (*v/v*) of HCl solution (1 M) was added to the plate, and after shaking it for 5 min, the absorbance was measured at 570 nm (reference at 630 nm) by the SpectraMax 340PC Absorbance Microplate Reader. All percentages of cell survival were calculated referring to the control.

### 3.8. Cell Fractionation, Isolation and Characterization of Lysosomes

For the evaluation of the endo/lysosomal escape of saporin-HRP via cellular fractionation of cells previously treated with the same conjugate in the presence of SA1641, ECV-304 cells were seeded in 75-cm^2^ dishes and grown to confluence in DMEM supplemented with 10% FBS and 1% PS. Cells of 4 dishes (1.5 × 10^7^ cells) were treated with 2 mL saporin-HRP (100 nM in medium) in the presence or absence of SA1641 (final concentration of 5 µg/mL) at 37 °C for 6 h. Cells were washed 3 times with 2 mL ice-cold PBS. Subsequently, 1 mL ice-cold PBS was added, and cells were scraped (Falcon Cell Scraper, Fisher Scientific/Thermo Scientific, Waltham, MA, USA) from the cell culture dishes. Furthermore, 1 mL ice-cold PBS supplemented with protease inhibitor Complete (Roche Applied Science, Mannheim, Germany) was added to re-suspended cells. Cells were loaded into a 2-mL cell homogenizer with a small clearance pestle (Sigma-Aldrich, Steinheim, Germany) and homogenized in 28 cycles. Initially, the homogenate was centrifuged at 1000 × *g* for 10 min at 4 °C. Then, the pellet was discarded, and the supernatant was further centrifuged at 100,000 × *g* and 4 °C for 1 h with the Optima L-90K Ultracentrifuge (Beckman Coulter, Krefeld, Germany). After ultracentrifugation, the supernatant corresponded to the (1) cytosolic fraction, while the pellet contained all cellular organelles, including the lysosomes. The pellet was re-suspended, frozen and thawed 5 times in order to disrupt the lysosomal membranes and centrifuged again at 100,000 × *g* and 4 °C for 1 h. After this second ultracentrifugation step, the supernatant corresponded to the (2) lysosomal fraction. The presence of saporin-HRP in both the (1) cytosolic fraction and (2) lysosomal fraction was analysed by the determination of peroxidase activity (see Section 3.6).

For the isolation of lysosomes to further characterize the endo/lysosomal escape of saporin-HRP in isolated organelles, eight 75-cm^2^ dishes with ECV-304 cells were allowed to proliferate to confluence (3 × 10^7^ cells) in DMEM supplemented with 10% FBS and 1% PS. After a washing step with PBS, cells were detached using 0.25% Trypsin-EDTA. Re-suspended cells were centrifuged for 5 min at 800 × *g* and 4 °C and washed with 10 mL ice-cold PBS. Cells were centrifuged and washed one more time and finally re-suspended in 6 mL ice-cold PBS. Cells were homogenized in 28 cycles by a 7-mL cell homogenizer with a small clearance pestle (Sigma-Aldrich, Steinheim, Germany). To achieve a crude fraction rich in lysosomes, the homogenate was submitted to differential centrifugation at 1000 × *g* for 10 min at 4 °C and the corresponding supernatant at 20,000 × *g* for 20 min. Ice-cold translocation buffer (400 µL) (20 mM HEPES, 10 mM MgSO_4_ and 0.1 M KCl, pH 7.0) supplemented with 2 mM adenosine triphosphate (ATP) was added to the pellet (obtained after centrifugation at 20,000 × *g*) that contained the lysosomes. The crude lysosomal fraction was stored at 4 °C.

Firstly, the permeabilizing effects of digitonin (Ysat, Wernigerode, Germany), α-hederin (Sigma-Aldrich, Steinheim, Germany) and SA1641 were measured on lysosomal membranes by the β-*N*-acetylglucosaminidase (NAG) assay [30]. The crude lysosomal fractions were shortly centrifuged at 1000 × *g* for 2 min at 4 °C to discard the aggregated organelles. Then, 5 µL of the crude lysosomal fraction was treated with 45 µL translocation buffer in the presence of digitonin, α-hederin or SA1641 at the final concentrations of 0.05, 0.5, 5, 10 and 50 µM at 37 °C for 1 h. To determine the amount of lysosomal enzyme NAG released from the lysosomes under these conditions, the lysosomes were centrifuged at 20,000 × *g* for 20 min, and β-*N*-acetylglucosaminidase activity in the supernatant (10 µL) was evaluated by the addition of a substrate solution (90 µL) (0.09 M citrate buffer, 1 mg/mL 4-nitrophenyl *N*-acetyl-β-d-glucosaminide, pH 4.7) previously equilibrated at 37 °C. The reaction was incubated at 37 °C for 10 min, and after the addition of 200 µL Na_2_CO_3_ (0.4 M), the absorbance was measured at 405 nm by the SpectraMax 340PC Absorbance Microplate Reader.

Secondly, for the evaluation of the endo/lysosomal escape of saporin-HRP in isolated lysosomes from cells previously treated with the same conjugate, ECV-304 cells were incubated with medium containing saporin-HRP (100 nM). After 6 h of the addition of the compound, cells were washed with PBS, and the detachment of cells was initiated by the addition of 0.25% Trypsin-EDTA. The procedure to isolate the lysosomes (in this case, loaded with saporin-HRP) and to obtain the crude lysosomal fraction was carried out as described previously. Then, the crude lysosomal fractions (15 µL) were incubated with 55 µL translocation buffer in the presence of the same saponins as before (digitonin, α-hederin or SA1641) at the final concentrations of 0.5, 5, 10, 20, 30, 40 and 50 µM at 37 °C for 1 h. Lysosomal suspensions were centrifuged at 20,000 × *g* for 20 min, and the amount of saporin-HRP present in the supernatants (released from the lysosomes) was determined by measuring the peroxidase activity (see Section 3.6).

### 3.9. Live Cell Imaging and Single Endo/Lysosome Analysis

Live cell imaging was utilized to follow the endo/lysosomal escape of ^Alexa^saporin in the presence of triterpenoidal saponins. DMEM medium supplemented with 10% FBS and 1% PS containing 50,000 ECV-304 cells was added to a cell culture μ-Dish 35 mm, low (Ibidi, Martiensried, Germany) and cells were allowed to proliferate for 24 h. Thereafter, the medium was removed, and cells were treated with 800 μL medium in the presence of 1000 nM ^Alexa^saporin for 3 h. Cells were provided with 8 μL (1 mg/mL) Hoechst 33342 (Life Technologies, Carlsbad, CA, USA) 30 min before the end of the incubation time. Additionally, cells were treated with 1 μL CellMask (Invitrogen, Darmstadt, Germany) 5 min before the same incubation time finished. Cells were washed with live cell imaging solution (Invitrogen, Darmstadt, Germany) supplemented with 5 mM d(+)glucose. The washing step was repeated twice. Then, cells were covered with 800 μL live cell imaging solution, and cell culture dishes were fixed in a heating chamber to maintain the temperature at 37 °C and start the live cell imaging. Subsequently, SA1641 (final concentration of 10 µg/mL) was added to cells and the endo/lysosomal escape of ^Alexa^saporin was monitored for the following 3600 s. Thereafter, a high concentration of SA1641 (final concentration of 80 µg/mL) was provided to the cells, and the experiment was finished after observing its short-term effects.

Cells were visualized by laser scanning microscopy (LSM780, Axio Observer Z1, Carl Zeiss, Jena, Germany). The microscope was equipped with a Plan-Apochromat 63×/1.40 Oil objective, and focus stability was assured by Definite Focus (Carl Zeiss, Jena, Germany). Images were acquired by the software, ZEN 2010 (Carl Zeiss, Jena, Germany).

For the single endo/lysosome analysis, cells were monitored for 7200 s after the addition of SA1641 (final concentration of 10 µg/mL). Endo/lysosomes that were constantly appearing in the images during the whole process of ^Alexa^saporin escape were localized, and regions of interest (for each single endo/lysosome) were defined using the software ImageJ (version 1.46r, NIH, USA). Regions of interest comprised the surroundings of the endo/lysosomes, but excluded the vesicle itself. Single endo/lysosome analysis was monitored from 2400 s to 4200 s after the addition of triterpenoidal saponin.

## 4. Conclusions

Three different reporters were studied for their ability to detect the endo/lysosomal escape of protein-based therapeutics. The reporters consisted of: (1) horseradish peroxidase (HRP), a protein presenting peroxidase activity; (2) Alexa Fluor 488 (^Alexa^), a small fluorophore emitting fluorescence; and (3) ricin A-chain (RTA), a toxin exhibiting cytotoxicity. The following conjugates were synthesized and characterized *in vitro*: saporin-HRP, ^Alexa^saporin and saporin-KQ-RTA. While HRP failed in reporting the endo/lysosomal escape of saporin in the presence of structurally-specific triterpenoidal saponins, Alexa Fluor 488 efficiently reported the endo/lysosomal escape of the toxin at a concentration of 1000 nM. Furthermore, Alexa Fluor 488 permitted the evaluation of the endo/lysosomal escape in a single endo/lysosome analysis. The usage of RTA as a reporter allowed detecting the process of endo/lysosomal escape with sensitivity down to 10 nM. The combination of the two successful reporters may act as a complementary technique to monitor the endo/lysosomal escape of a protein-based therapeutic at high protein concentrations (1000 nM with Alexa Fluor 488 reporter), as well as at concentrations in the low nanomolar range (10 nM with RTA reporter). The developed methods would be surely of great interest not only in toxin-based therapeutics, but also in the case of other protein therapeutics, wherein a reporter for the proper assessment of its endosomal escape is warranted. This would also facilitate a proper assessment of effective therapeutic concentrations, at least during *in vitro* assessment.

## Supplementary Files

Supplementary File 1 Supplementary Information (ZIP, 17084 KB)

## Abbreviations

^Alexa^: Alexa Fluor 488
DMEM: Dulbecco’s modified Eagle’s medium
FBS: Foetal bovine serum
HRP: Horseradish peroxidase
MTT: 3-(4,5-Dimethylthiazol-2-yl)-2,5-diphenyltetrazolium bromide
NTA: Nitrilotriacetic acid
PS: Penicillin/streptomycin
RIP: Ribosome-inactivating protein
RTA: Ricin A-chain
