# Supplementary material for: Reporter Assay for Endo/Lysosomal Escape of Toxin-Based Therapeutics
Source: Toxins (Basel). 2014 May 22;6(5):1644–66. doi: 10.3390/toxins6051644 (PMC4052257; doi:10.3390/toxins6051644)
Supplement: Supplementary File 1 — Supplementary Information (ZIP, 17084 KB) [file toxins-06-01644-s001.zip › toxins-53644-Supplementary.pdf]

Supplementary information

Figure S1. Purification of saporin by Ni-NTA chromatography. Proteins were eluted with increasing concentrations of imidazole (31, 62, 125 and 250 mM). The flow-through, wash and all elution fractions were analyzed by SDS-PAGE. Saporin was eluted from Fraction 1 at 62 mM to Fraction 5 at 125 mM imidazole. Fractions comprising of only saporin but no or minor amount of co-purified proteins [Fractions 3–5 (62 mM) and Fractions 1–5 (125 mM)] were used for the further experiments.

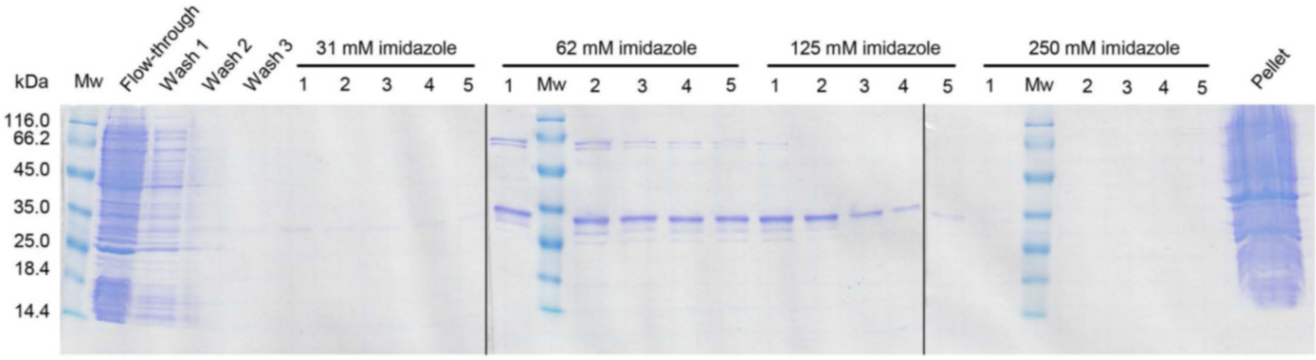

Figure S2. Purification and identity validation of the enzymatically inactive variant of saporin (saporin-KQ). (**A**) Purification of saporin-KQ by Ni-NTA chromatography. Elution fractions at 31, 62, 125 and 250 mM imidazole were assessed by SDS-PAGE. Saporin-KQ was eluted from Fraction 2 at 62 mM to Fraction 3 at 125 mM imidazole. Fractions comprising of only saporin-KQ but no or minor amount of co-purified proteins [Fractions 2–3 (125 mM)] were used for the further experiments; (**B**) Identity validation of saporin-KQ. Firstly, saporin-KQ (fraction from Ni-NTA chromatography with co-purified proteins) was analyzed by SDS-PAGE. Saporin and a pellet of the bacteria that had previously expressed saporin-KQ were simultaneously analyzed as controls. Secondly, saporin-KQ was analyzed by Western blot with a primary polyclonal antibody against saporin. Saporin-KQ, as well as saporin and saporin-KQ in the bacterial pellet, were specifically detected.

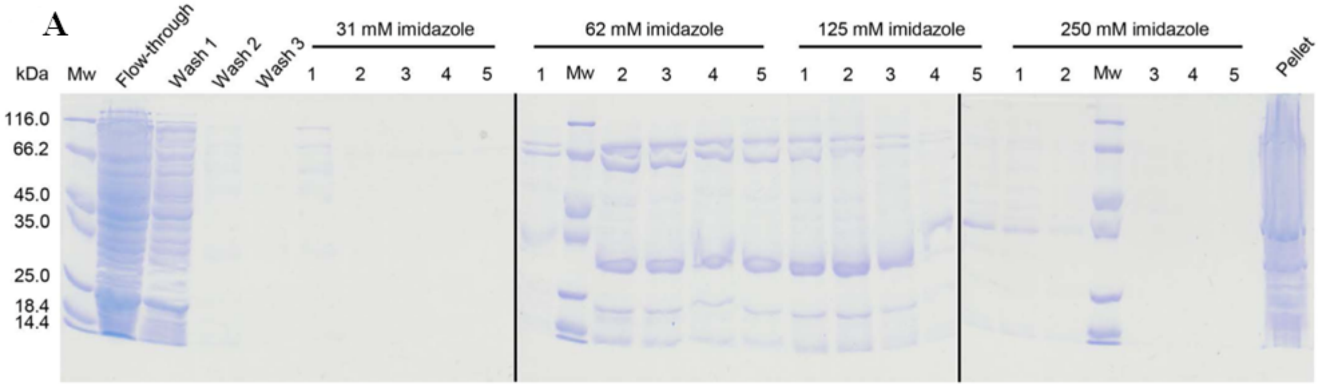

Figure S2. *Cont.*

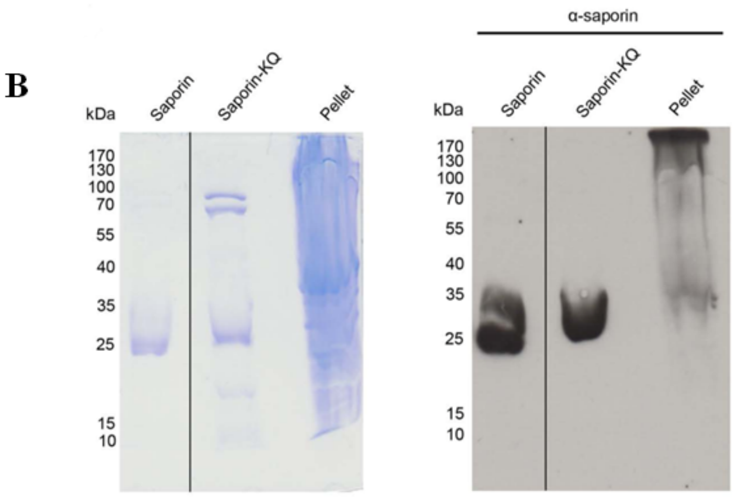

Video S1. Alexa Fluor 488 as a reporter for endo/lysosomal escape in live cell imaging. The same results presented in Figure 3 are shown here in video format. The video comprises all the fluorescence images acquired during the whole experiment (from t = 0 s to t = 3700 s).
